# Machine perfusion of the liver and in vivo animal models: A systematic review of the preclinical research landscape

**DOI:** 10.1371/journal.pone.0297942

**Published:** 2024-02-08

**Authors:** Wenjia Liu, Decan Jiang, Mareike Schulz, Constança Figueiredo, Daniele Dondossola, Franziska Alexandra Meister, Dora Krisztina Tihanyi, Arianeb Mehrabi, Rene Hany Tolba, Zoltan Czigany, Lisa Ernst

**Affiliations:** 1 Department of Surgery and Transplantation, Faculty of Medicine, University Hospital RWTH Aachen, Aachen, Germany; 2 Institute for Laboratory Animal Science and Experimental Surgery, Faculty of Medicine, RWTH, Aachen International University, Aachen, Germany; 3 Institute of Transfusion Medicine and Transplant Engineering, Hannover Medical School, Hannover, Germany; 4 General and Liver Transplant Surgery Unit, Fondazione IRCCS Ca’Granda Ospedale Maggiore Policlinico, Milan, Italy; 5 Doctoral School of Clinical Medicine, Semmelweis University, Budapest, Hungary; 6 Department of General, Visceral and Transplantation Surgery, Heidelberg University Hospital, Heidelberg, Germany; Hospital Israelita Albert Einstein, BRAZIL

## Abstract

Machine perfusion (MP) is often referred to as one of the most promising advancements in liver transplantation research of the last few decades, with various techniques and modalities being evaluated in preclinical studies using animal models. However, low scientific rigor and subpar reporting standards lead to limited reproducibility and translational potential, hindering progress. This pre-registered systematic review (PROSPERO: CRD42021234667) aimed to provide a thematic overview of the preclinical research landscape on MP in liver transplantation using in vivo transplantation models and to explore methodological and reporting standards, using the ARRIVE (Animal Research: Reporting of In Vivo Experiments) score. In total 56 articles were included. Studies were evenly distributed across Asia, Europe, and the Americas. Porcine models were used in 57.1% of the studies, followed by rats (39.3%) and dogs (3.6%). In terms of graft type, 55.4% of the studies used donation after cardiac death grafts, while donation after brain death grafts accounted for 37.5%. Regarding MP modalities, the distribution was as follows: 41.5% of articles utilized hypothermic MP, 21.5% normothermic MP, 13.8% subnormothermic MP, and 16.9% utilized hypothermic oxygenated MP. The stringent documentation of ARRIVE elements concerning precise experimental execution, group size and selection, the choice of statistical methods, as well as adherence to the principles of the 3Rs, was notably lacking in the majority of publications, with less than 30% providing comprehensive details. Postoperative analgesia and antibiotics treatment were not documented in 82.1% of all included studies. None of the analyzed studies fully adhered to the ARRIVE Guidelines. In conclusion, the present study emphasizes the importance of adhering to reporting standards to promote reproducibility and adequate animal welfare in preclinical studies in machine perfusion. At the same time, it highlights a clear deficiency in this field, underscoring the need for further investigations into animal welfare-related topics.

## Introduction

Machine perfusion (MP) is considered one of the most promising recent technical advancements in the field of solid organ transplantation research [1]. Keeping organs alive outside the human body using artificial systems has already fascinated brilliant scientists such as the Nobel laureate Alexis Carrel more than a century ago [2].

Following extensive research and development, including preclinical animal studies and testing commencing in the late eighties, liver MP entered the clinical arena a decade ago and reached its prime time with three major randomized controlled trials (RCTs) published over the past three years with two of these reported just very recently [3–12].

A wide range of machine perfusion devices and modalities are currently being used for the preservation, reconditioning, and viability assessment of donor allografts [1, 4]. *Ex situ* MP can also provide a unique platform to deliver targeted allograft therapies with multiple benefits, such as the prevention of systemic or off-target effects in the donor and recipient, as well as reduced costs [13]. Although viability assessment and allograft therapeutics will certainly have a meaningful clinical impact in the field of organ transplantation in the near future, they are still in their infancy of clinical translation with a plethora of unanswered questions; this calls for powerful animal models and tools to generate high-quality preclinical evidence with translational potential.

Experimental organ transplantation has provided the basis for almost every scientific or technical advancement in the field of solid organ transplantation [14]. Over the last seven decades, experimental liver transplantation (LT) was widely used to investigate the pathophysiological and immunological responses of the human body to LT as well as to develop new surgical techniques, approaches of organ preservation and novel immunosuppression drugs [14]. Although animal experiments were inevitable in LT research, the traditional “bench-to-bedside” approach of clinical translation is being increasingly criticized, both in the scientific and popular press [15–17]. The critical voices are often related to the low scientific rigor, inferior reporting standards, reproducibility and associated limitations in the translational potential [18].

On the analogy of the widely recognized CONSORT (Consolidated Standards of Reporting Trials) statement for clinical trials, researchers of the NC3Rs (National Centre for the Replacement and Reduction of Animal Experiments) developed and introduced the ARRIVE guidelines published over ten years ago, aiming at improving the reproducibility of biomedical research using animal models. Since published in 2010, more than 600 journals have endorsed its use until 2016 [19, 20].

This systematic review aims to provide not just a thematic overview of the available preclinical literature on MP science in LT using *in vivo* transplantation models but also explores methodical and reporting standards for the first time in this field.

## Materials and methods

This preclinical systematic review with non-interventional outcome measures has been performed according to the principles of the Preferred Reported Items for Systematic Review and Meta-analyses (PRISMA) guidelines as far as applicable (S2 and S3 Files) [21]. Details of the *ex-ante* study protocol for this systematic review were registered open access on PROSPERO (Registration Number: CRD42021234667. S4 File) [22].

### Search strategy

A comprehensive and systematic literature search was performed in the following electronic bibliographic databases: PubMed, Web of Science, and Embase, with no time restrictions and additional search filters. The complete search strategy was based on the main search terms of liver, liver transplant, hepatic transplant, liver transplantation, machine perfusion, and machine preservation; searching details are available from S3 Table. According to the Cochrane recommendations, we did not limit the search terms to title, abstract or keywords, resulting in the inclusion of MeSH and Emtree as well as related terms [23]. The systematic literature search was carried out in March 2021, encompassing literature ranging from 1990 to February 2021 and lately updated Aug. 2023. Results were imported to Rayyan software for title/abstract screening [24]. The literature search was performed independently by three investigators (WL, DJ and MS). Disagreements concerning the suitability of articles were discussed and decided by the core study team (WL, DJ, ZC, MS) through which a consensus was reached. Subsequently, full texts of potentially suitable articles were retrieved and assessed based on the eligibility criteria. If applicable, online supplementary files were also retrieved for further analysis.

### Inclusion criteria and exclusion criteria

Inclusion and exclusion criteria were predefined and described in detail in our registered study protocol [22]. Inclusion criteria: (1) English language original research publications in international peer-review literature; (2) living animal models using liver transplantation; (3) machine perfusion as the organ preservation method or study intervention.

For further selection, (1) systematic reviews and meta-analyses of experimental studies as well as case studies were excluded. (2) All studies in which the liver is only retrieved but no transplantation procedure was performed, thus the liver was not implanted into the same (auto-transplantation) or to a different recipient animal, and (3) full-text publication in any other language than English was likewise excluded.

### Data extraction and analysis

We performed a comprehensive synthesis of the current scientific literature on preclinical MP using descriptive statistics and bibliometric methods. All data were extracted into a study-specific spreadsheet database. The following paper characteristics and data were extracted: publication activity (1990–1999, 2000–2009, 2010–2019, 2020-2023/January; pre-ARRIVE, <2010, post-ARRIVE, >2010); geographic area (numbers of articles from Europe, Americas, Asia and each country); journal impact factor (IF) in the year of publication estimated over 2 years; journal category rank, source: https://www.scimagojr.com; total citations; type of the institution (academic clinical department, academic research department, research institute, hospital); number of funding sources and source of funding; species and strain of experimental animal; sex; transplantation model (allogenic or syngenic model /autotransplant); donor type; graft size and graft flush; applied machine perfusion protocol; most frequently applied maximum follow up; immunosuppression; analgesia postoperative; relationship of cooperation between countries; average number of articles; ARRIVE score; funding; IF; citations per year estimated over 2 years source https://www.scijournal.org; and MP parameters (perfusion time, temperature, solution, pressure). A quality assessment of all selected articles was performed according to the ARRIVE score adapted from a previous study of our group [20]. The ARRIVE guideline checklist contains a scoring system of 20 main items for reporting quality. For details on the grading system, see S1 Table. Briefly, a predefined grading (e.g., 0 = clearly insufficient; 1 = possibly sufficient; and 2 = clearly sufficient) was applied for all ARRIVE items, and the sum of scores has been expressed as ARRIVE score with a maximum sum of 78 (S1 Table). The quality scoring was performed independently by three authors (WL, DJ, and MS).

Cohen’s kappa was used to assess inter-observer agreement. A k > 0.5 was considered moderate, and k > 0.8 was high consistency. Subgroup analysis was compared by the Mann-Whitney U and Kruskal-Wallis H tests, as applicable. A p-value of <0.05 was considered significant. Worldwide distribution of the included articles has been created with Biorender.com (license #EF266S0Z6W). InteractiVenn was applied for the distribution of papers using various MP modalities [25]. IBM SPSS Statistics 26 for Windows (IBM Inc, Armonk, NY) was used for statistical analysis, and further data were plotted by the Prism Graph Pad for Windows version 9.4.1 software (GraphPad Software Inc, La Jolla, CA). Data are given as mean and standard deviation unless otherwise stated. Each data set was tested for normality before applying the t-test or One-Way ANOVA.

## Results

### Study selection

**Fig 1:**
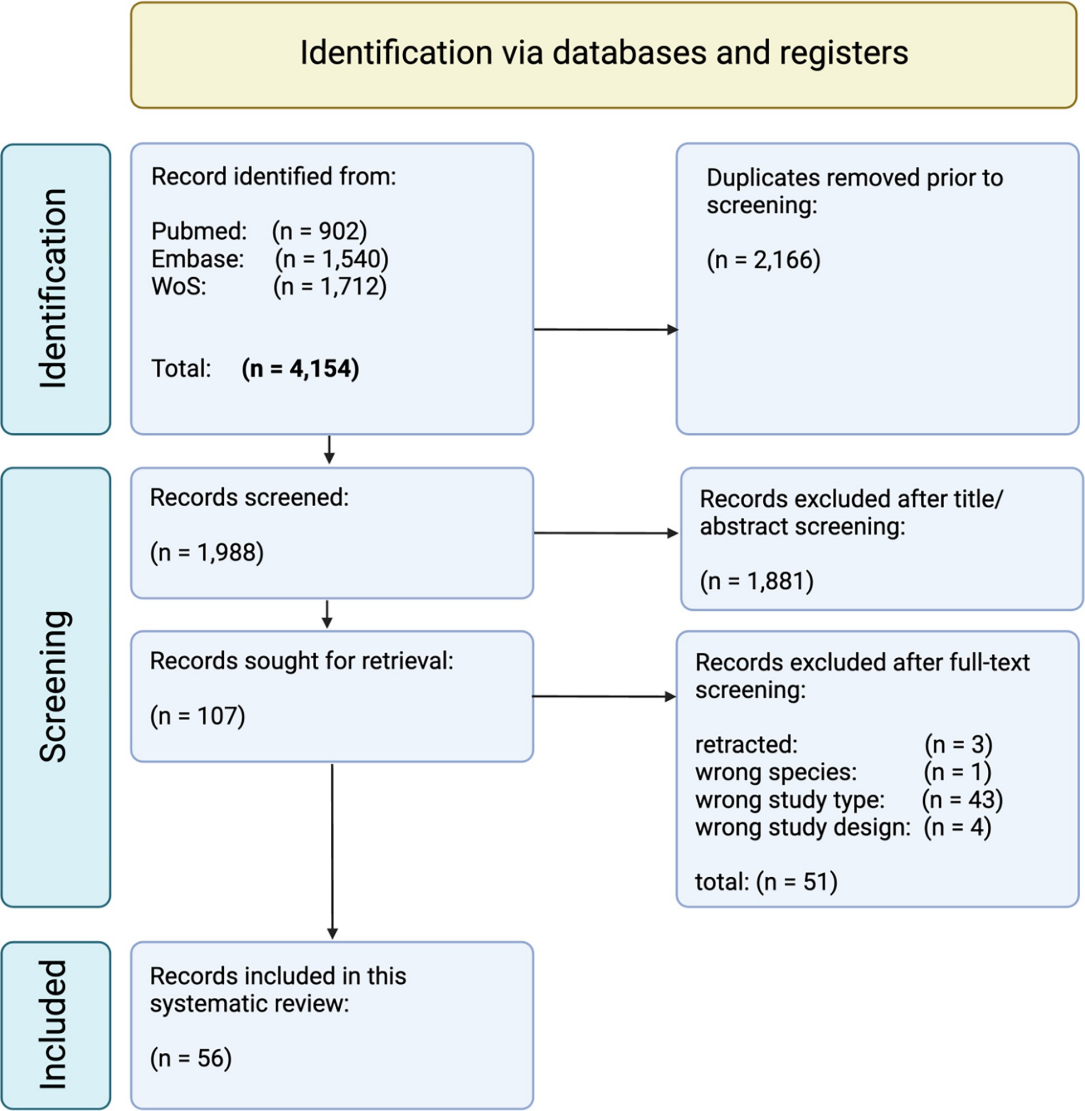
Flow diagram based on the PRISMA recommendations (2020).

A total of 4,154 potentially relevant articles were initially identified from the three databases PubMed, Web of Science and EMBASE. Fig 1 depicts the flow diagram based on the PRISMA recommendations [21]. After elimination of duplicates and papers published in other languages than English 1,988 papers remained acceptable for further investigation. Excluding 1,881 not-related articles resulted in 107 studies eligible for the full-text screening phase. Some 51 articles were excluded after full-text screening was performed. Meeting the inclusion and exclusion criteria, 56 articles were identified as eligible for final analysis. Cohen’s kappa showed a substantial agreement between the investigators’ WL, DJ and MS with a value ϰ = 0.73 for the selection of the final cohort of papers. The complete list of supporting references can be found in the S2 Table.

### Characteristic information

The total period of this review covers about 33 years, dating from the first hit in 1990 to the last update in August 2023. Table 1 shows all major characteristic data of the included studies grouped into significant categories. We summarized results of the 56 articles in major research characteristics: (1) development over time, (2) animal species, (3) model characteristics, (4) geological distribution, (5) academic network and (6) modalities of MP (S1 File).

**Table 1:** Characteristics of the 56 included studies.

Category	Characteristic	Number (% of total n = 56)
**Publication activity **	1990–1999	3 (5.4)
	2000–2009	10 (17.9)
	2010–2019	32 (57.1)
	2020–2023	11 (19.6)
	Pre-ARRIVE	13 (23.2)
	Post-ARRIVE	43 (76.8)
**Journal impact factor in the year of publication **	0–2	18 (32.1)
	2–5	22 (39.3)
	5–10	9 (16.1)
	10–20	1 (1.8)
	20-	0 (0)
	None	6 (10.7)
**Journal category rank in the year of publication **	Q1	30 (53.6)
	Q2	6 (10.7)
	Q3	16 (28.6)
	Q4	1 (1.8)
	None	3 (5.4)
**Total citation **	0–10	14 (25.5)
	11–50	22 (40)
	51–100	5 (9.1)
	101–500	14 (25.5)
**Regions (based on the affiliation of corresponding author) **	Europe	17 (30.4)
	Americas	17 (30.4)
	Asia	22 (39.3)
**Institutions **	Academic Clinical Department/ University Hospital	47 (78.3)
	Academic Research Department	5 (8.3)
	Research Institute	1 (1.7)
	Other	7 (11.7)
**Number of funding sources **	1	11 (19.3)
	2	7 (12.3)
	3	7 (12.3)
	4	6 (10.5)
	≥5	10 (17.4)
	Not clear/Not reported	16 (28.1)
**Source of funding **	Government	30 (40)
	Foundations	22 (29.3)
	Industry	7 (9.3)
	Not clear/Not reported	16 (21.3)
**Species and strain of the laboratory animals **	Rodent	22 (39.3)
	*Sprague-Dawley*	5 (20)
	*Lewis*	11 (44)
	*Brown Norway*	8 (32)
	*Fischer (F344)*	1 (4)
	Porcine	32 (57.1)
	*Large white*	1 (3.1)
	*Yorkshire*	4 (12.5)
	*Bama*	3 (9.4)
	*Landrace*	5 (15.6)
	*Other cross bred*	8 (25)
	*Not clear/Not reported*	11 (34.4)
	Canine	2 (3.6)
	*Mongrel*	1 (50)
	*Not clear/Not reported*	1 (50)
**Sex **	Male	25 (44.6)
	Female	8 (14.3)
	Both	5 (8.9)
	Not clear/Not reported	18 (32.1)
**Allogenic or syngenic model /autotransplant **	Allogenic	34 (60.7)
	Syngenic	15 (26.8)
	Auto-transplant	3 (5.4)
	Not clear/Not reported	4 (7.1)
**Donor Type (DCD/DBD) **	DCD	31 (55.4)
	DBD	21 (37.5)
	Both	4 (7.1)
**Graft Type **	Whole liver graft	54 (96.4)
	Partial/split liver	2 (3.6)
**Graft flush before MP **	Portal vein	18 (32.1)
	Arterial (Aorta or Hepatic artery)	3 (5.4)
	PV+AO/HA	20 (35.7)
	Not clear/Not reported or not performed	15 (26.8)
**Machine perfusion modality **	HMP	27 (41.5)
	HOPE	11 (16.9)
	SNMP	9 (13.8)
	NMP	14 (21.5)
	HNPE	2 (3.1)
	controlled rewarming (4–25°C)	1 (1.6)
	HMP and controlled rewarming (4–25°C)	1 (1.6)
**Most frequently applied maximum follow-up of the animals **	3 d	13 (36.1)
	5 d	10 (27.8)
	7 d	7 (19.4)
	28 d	6 (16.7)
**Immunosuppression **	Tacrolimus	8 (14.3)
	Methylprednisolone	1 (1.8)
	Cyclosporine	1 (1.8)
	Methylprednisolone and Cyclosporine	2 (3.6)
	Not clear/Not reported or not performed	44 (78.6)
**Analgesia postoperative **	Buprenorphine	7 (12.5)
	Morphine	2 (3.6)
	Carprofen	1 (1.8)
	Not clear/not reported	46 (82.1)
**Antibiotics postoperative **	Enrofloxacin	1 (1.8)
	Metronidazole	2 (3.6)
	Cephalosporine	4 (7.1)
	Amoxicillin	1 (1.8)
	Not clear/not reported or not performed	46 (82.1)

**Fig 2:**
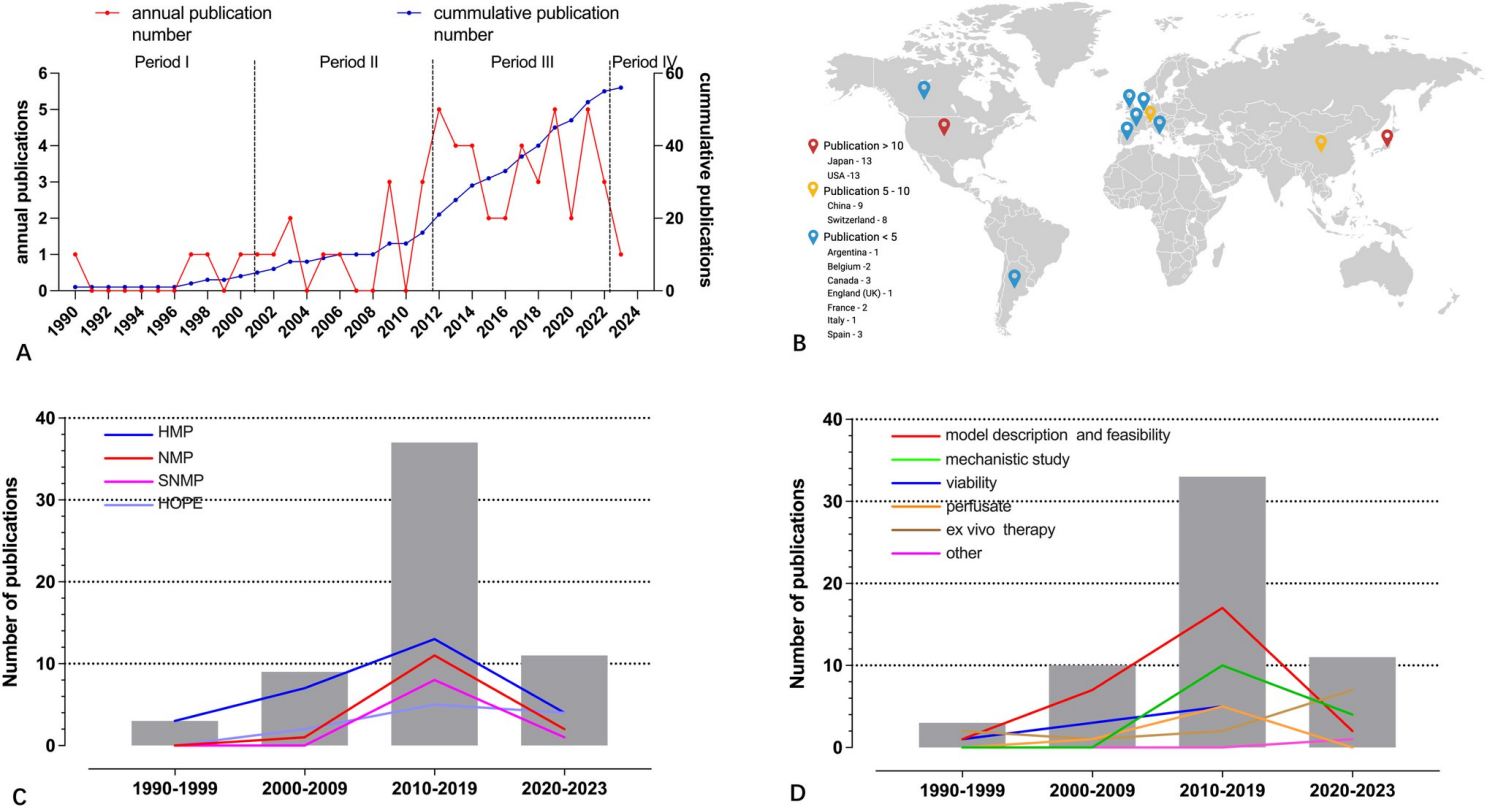
Publication characteristics (A). Worldwide distribution among the included 56 publications over the 33 years period (B). Distribution of machine perfusion publications (C). Distribution of main research topic (D). Bars represent cumulative number of studies per time period, subgroups are displayed with coloured lines (Fig 2C and 2D). Fig 2B was created with Biorender.com.

The number of publications using LT models and MP increased considerably over the last recent three decades, with 3 (5.4%), 10 (17.9%), 32 (57.1%) and 11 (19.6%) articles published between Feb.1990-1999 (period I), 2000–2009 (period II), 2010–2019 (period III) and 2020-Jan. (period IV) 2023 respectively (Table 1, Fig 2A). Before the first publication of the ARRIVE guidelines in 2010, only 13 (23.2%) articles were published.

In more than half of the studies (n = 32, 57.1%) porcine models were used, followed by rats (n = 22, 39.3%) and dogs (n = 2, 3.6%). Most frequently allogenic (n = 34, 60.7%), followed by syngenic (n = 15, 26.8%) models where used. An auto-transplantation models was used in 3 studies (5.4%), whereas 4 studies (7.1%) failed to report the transplantation model. Importantly, most studies, postoperative management, like analgesic (n = 46, 82.1%) and antibiotic treatment (n = 46, 82.1%) were not documented.

With the start of MP investigations in the late nineties, there was a nearly equal geographic distribution of the articles between Europe (n = 17, 30.4%), Americas (n = 17, 30.4%), and Asia (n = 22, 39.3%). An overview of the numbers of publications by country is shown in Fig 2B displaying the highest number of articles in Japan (n = 13) and the United States (n = 13), followed by China (n = 9), and Switzerland (n = 8).

Over the three decades, MP modalities were well established and there has been a marked decrease in the number of relevant studies. Nevertheless, we found a increasing trend in the area of ex vivo therapy (Fig 2D).

The ranking of the number of publications by journal is shown in Table 2 in which Transplantation Proceedings (n = 16) ranks first, followed by Transplantation (n = 8) and an equal distribution at Annals of Surgery and Plos One (each n = 4). Cooperation network in multinational studies was most expressed in papers from the United Kingdom (9 connections) and the United States (7 connections), followed by Spain (6 connections). Interestingly, countries are more or less interconnected of all the analysed countries except China, which had no external links (S1 Fig). The majority of authors were affiliated with academic clinical research institutions (n = 47, 78.3%), whereas academic research institutions without connection to any hospital were found in 5 cases. Only one independent research institute contributed to this systematic review (Table 1). The source of funding was described in more than two-third of all articles (n = 40, 71.9%). The studies were mostly supported by grants from various research foundations and/or by government (total n = 30, 40%), whereas only seven studies (9.3%) where funded solely by industry. Over 30 studies (52.6%) describe funding from two or more resources.

**Table 2:** Top six journals with the highest number of publications.

Journal	Publications	Mean IF	TC	Mean TC/ publications	
Transplantation Proceedings	16	1.003	490	30.6
Transplantation	8	4.099	497	82.8
Annals of Surgery	4	6.499	751	188
Plos One	4	3.365	95	23.8

IF: Impact Factor based on Mean of IF in the year of publication

TC: total citation, sum of included studies per journal

Journals were ranked based on the number of included publications

### Quality assessment according to the ARRIVE guidelines

**Fig 3:**
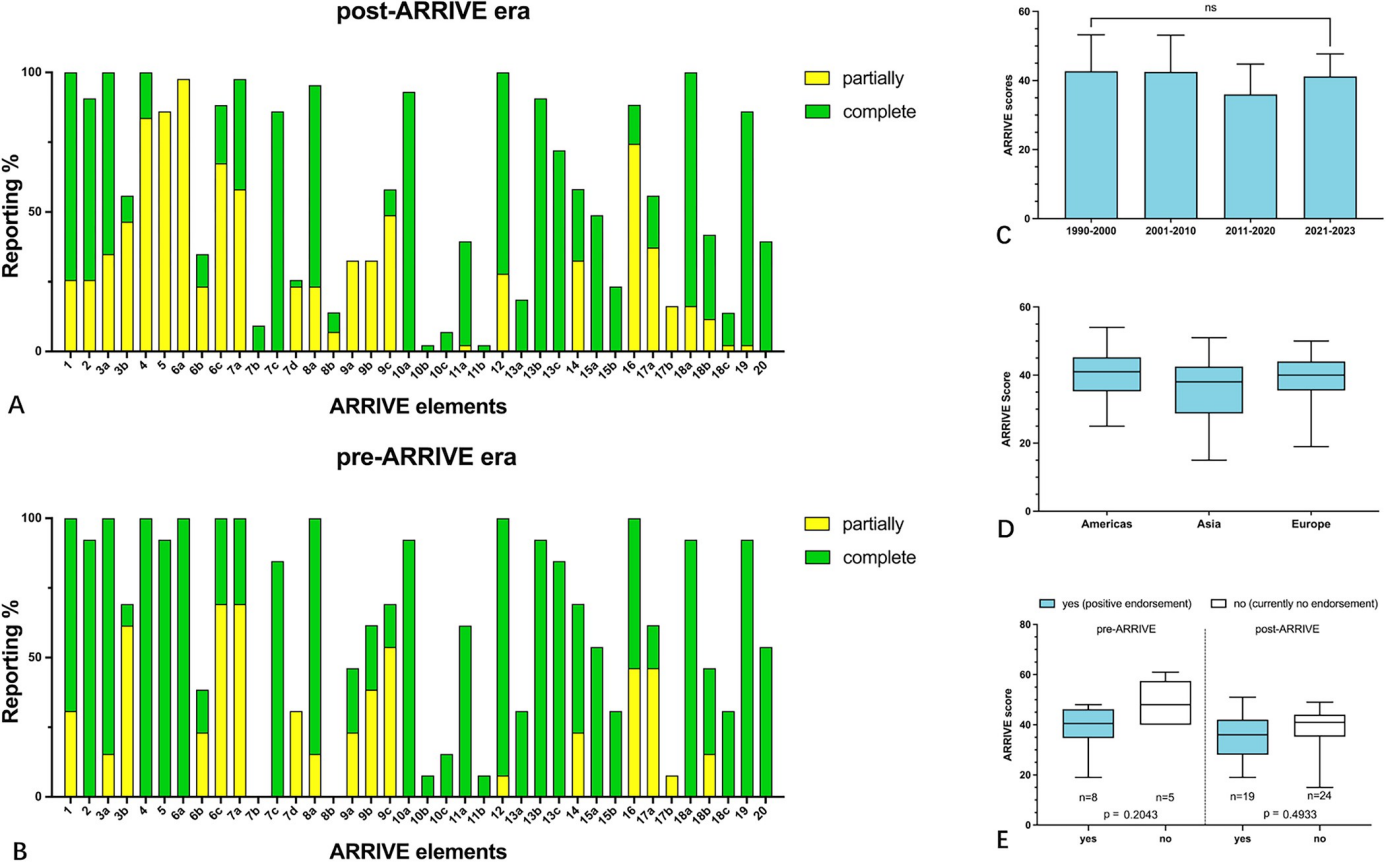
Percentage of the reporting of the various elements of the ARRIVE guidelines in the post- and pre-ARRIVE era (A, B). Subgroup analysis of the distribution of ARRIVE scores and publication period (C). ARRIVE scores and region (One-Way ANOVA, Kruskal-Wallis test) (D). Distribution analysis of ARRIVE scores and journals which official endorse the ARRIVE guidelines in the post-ARRIVE era. The One-Way ANOVA for parametric distribution showed no significance between the pre-ARRIVE (p = 0.2043) and the post-ARRIVE era (p = 0.4933) (E).

The maximum achievable score according to the ARRIVE Guideline checklist was 78. However, this was not reached by any of the analysed studies. The mean ARRIVE score of the included papers was 38.4 ± 9.1. Fig 3A depicts the reporting of each single ARRIVE element in the pre- / post-ARRIVE era expressed in percentage and partly or full adherence. In all items except 7b, 8b, 17b and 18a (when (time of day), provide animals information, adverse events, and interpret the results) the overall frequency of reporting the respective information improved over time. The least frequently reported ARRIVE elements (reported in <30% of the articles) were 7b, 7d, 8b, 10b, 10c, 11b, 13a, 15b, 17b, and 18c. These items represent details of the study design e.g., sources of experimental animals, sample size calculation, replication and distribution, as well as statistical methods and numbers, describe modifications to the experimental protocols made to reduce adverse events, and impact on the implementation of the 3R (Reduction, Replacement, Refinement) of animal experiments.

Table 3 lists the 5 top increases in ARRIVE elements over the past years. Following the ARRIVE guidelines, the report of funding resources (Item 20) was introduced for the first time as a required reporting element and is now included in 34 of 43 studies (79.1%) of all publications (pre-ARRIVE era 6 of 13 (46.2%).

**Table 3:** Top five items improved the most in reporting standards.

Item	Item description	PreARRIVE-PostARRIVE
9b	Husbandry conditions	31.8%-61.5%
11a	Give full details of how animals were allocated to experimental groups	38.6%-61.5%
18c	Implications of your experimental methods or findings for the replacement, refinement or reduction of the use of animals in research	13.6%-30.8%
20	Funding- List all funding sources (including grant number) and the role of the funder(s) in the study	38.6%-53.8%
3b	Explain how and why the animal species and model being used can address the scientific objectives and, where appropriate, the study’s relevance to human biology	54.5%-69.2%

Analysis of the distribution of the ARRIVE score based on the publication period, regions, and ARRIVE score according to journals which do or do not endorse the guidelines, is shown in Fig 3C–3E. However, there is no significant change between the time periods when comparing the total ARRIVE score. There is also no significant difference between studies from Americas, Asia or Europe in adherence to the ARRIVE guidelines.

Journals which officially endorse the ARRIVE guidelines are listed at https://arriveguidelines.org/supporters/journals. A comparison of included studies sorted by this endorsement and separated in pre- and post-ARRIVE era showed no significance between both groups regardless of the era (Fig 3E). Additionally, we could not show a significant difference between pre- and post-ARRIVE era of journals which endorse the ARRIVE guidelines (p = 0.7436).

An analysis of the number of funding sources compared to the ARRIVE score revealed no correlation (R^2^ = 0.0002). Number of citations a paper received had no significant association with the reporting quality of the manuscript assessed by the ARRIVE score (p = 0.2712).

### Machine perfusion modalities

**Fig 4:**
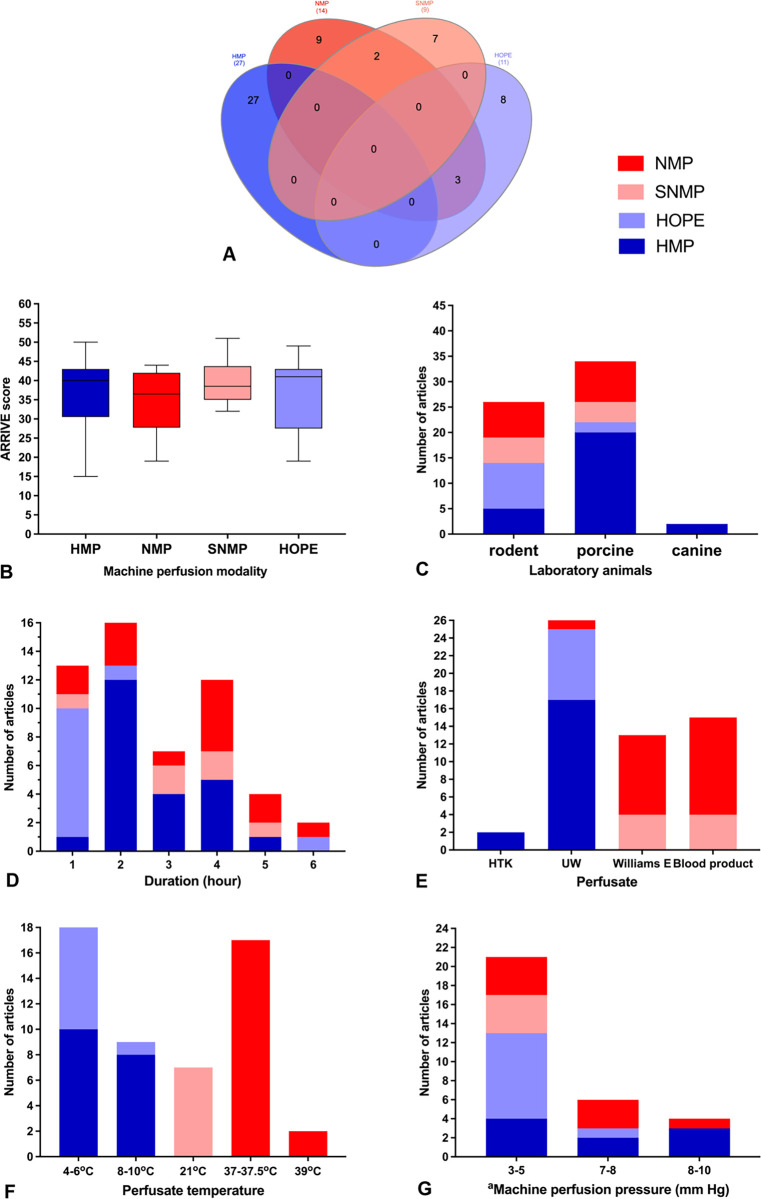
Venn diagram of the used machine perfusion modalities and combined studies studying more than one modality (A). Analysis of the distribution of ARRIVE scores and machine perfusion modality (B). Visualization of laboratory animals, machine perfusion characteristics and conditions used in the 56 studies (C, D, E, F, and G). ^a^Represents portal vein pressure.

Fig 4 shows a Venn diagram (Fig 4A) of the different modalities and combinations of machine perfusion techniques and their characteristics (Fig 4C–4G) used in the analyzed dataset. In this review, NMP, SNMP, HOPE and HMP for liver perfusion were included. The technique of HMP (n = 27) was used the most, followed by NMP (n = 14), HOPE (n = 11) and SNMP (n = 9). The publications using various MP modalities achieved a similar overall ARRIVE Score, without significant difference (Fig 4B). The majority of the included studies focused on pig models, followed by rat models and occasionally canine models, with a particular emphasis on porcine models while using the HMP techniques (Fig 4C). Nevertheless, a relatively equal application of the various MP modalities was found in the rat model. The duration of liver perfusion differs in the various modalities between one hour to a maximum of six hours. The majority of the studies used 2-hour perfusion in the HMP setting whereas, in the NMP setup, a four-hour preservation time was the most frequent. Utilization of the individual perfusates is primarily based on the modality: For example, Histidine-Tryptophan-Ketoglutarate Solution (HTK) has only been used in a limited number of HMP studies, while University of Wisconsin solution (UW) perfusate has been used in both hypothermic and normothermic techniques, making it the most frequently used perfusate in the studies analysed, accounting for twenty-eight of the total studies (Fig 4E). Williams E perfusate as well as blood containing solutions are only used in the warmer normo- or subnormothermic settings. Temperature categories shown in Fig 4F are referring to the characteristic conditions of the respective modality. Hypothermic techniques like HMP and HOPE refer to a temperature below 10°C. Here, the majority of the publications (n = 18) were reporting a perfusion temperature of 4–6°C, while nine studies reported 8–10°C. Twenty-one°C was the standard temperature for SNMP, while the normothermic perfusion was either conducted at body temperature range of 37 to 37.5°C of all the studies. Particularly, two articles were found using perfusion modalities with up to 39°C. The pressure values during liver perfusion are displayed in Fig 4G indicating a low-pressure system of max. 5 mmHg portal venous pressure used across all modalities.

## Discussion

This study reports the results of a systematic review of all preclinical animal studies using *in vivo* models of LT in combination with MP covering the last three decades. A comprehensive analysis of the reporting quality of 56 included studies was conducted. The analysis utilized the ARRIVE score previously described [21] to assess the quality of reporting.

Improvements in surgical technology, perioperative management, and immunosuppressive therapy substantially enhanced clinical outcomes of LT recipients over the past decades [26–28]. However, organ shortage and the increasing use of marginal or extended criteria donor allografts (ECD) call for continued innovation in the field of allograft preservation.

The full complexity of the pathophysiological responses and mechanistic processes during LT can only be replicated and studied properly including in vivo models using living animals. Therefore, this study focuses exclusively on *in vivo* LT models.

Machine perfusion technology is an alternative to static cold storage being at the forefront of innovation in the last decades [29]. Although MP was used during the early pioneering years of LT, it was subsequently abandoned in favour of the cheap and simple static cold storage technique, which has dominated the field for the past 40 years [29]. In the past decades, different modalities of machine perfusion have been developed, including Hypothermic Machine Perfusion (HMP), Normothermic Machine Perfusion (NMP), Subnormothermic Machine Perfusion (SNMP), and Hypothermic Oxygenated Perfusion (HOPE). In general, the use of cold and warm perfusion techniques was found in this review to be balanced in preclinical applications.

Experimental studies and in vivo animal models are still essential to investigate the subcellular protective mechanisms, safety and feasibility of various machine perfusion modalities and new devices [30]. In vivo testing can therefore be designated as inevitable to investigate various on-pump therapeutic allograft interventions which are increasingly in the spotlight of interest. According to the observations of our systematic review, a major part of such MP studies was published in recent years with a gradually increasing publication activity over the whole observation period with a well-balanced distribution of studies between Asia, Europe, and the Americas (Table 1). However, an exceptionally strong publication activity was observed from the US, Japan, and China. Interestingly, in contrast to previous studies based on other research topics where small animal models, especially rodents dominate the field, in our present report a high number of the included studies were carried out in large animals, mostly in porcine models. This trend might be attributed to the steep learning curve of the microsurgical techniques required to master small animal models of LT and the greater translational significance and comparability with humans when using large animals. The included studies showed a wide range of heterogeneity in graft types (DBD/DCD models, split/reduced size vs. whole liver transplants, and allogenic, syngenic and auto-transplant models). Additionally, they evaluated the various machine perfusion modalities with their respective characteristics, indicating that animal models cover the entire spectrum of preclinical MP research.

Ground-breaking LT research using animals may still face challenges due to insufficient knowledge about animal models, increasing awareness of animal welfare, poor study design, conduction, analysis, and reporting. This may result in project failure or leading to incorrect conclusions [36]. There is evidence that less than one-third of animal-based research is translated to human trials [31, 32]. This stresses the importance of the need for well-designed and sufficient-reported animal studies. Therefore, the ARRIVE guidelines aimed to ensure the completeness and transparency of animal studies to maximize the output from research using laboratory animals and gain reproducibility [33]. In our systemic review we do not only give a thematic overview of the available preclinical literature on MP in LT using in vivo transplantation models but also aim to explore methodical and reporting standards. To the best of our knowledge, this is the first systematic review in the topic of LT field with this particular focus, with the largest dataset spanning over a 33-year period.

Among 4,154 records screened, only 56 of them were finally included. Most of the studies were eliminated because MP techniques were performed in *ex vivo* setting without the procedure of implantation and follow-up in vivo. This can be attributed to the fact that the LT technique in animals is technically challenging [34, 35]. The need for a specialized skillset may be further underlined by the data, showing that 78.3% of these studies were conducted in an academic clinical department, rather than the academic research department (8.3%) or research institutes (1.7%). Detailed quality assessment of the eligible literature was performed based on the 20-items ARRIVE checklist [33]. According to the analysis, publication activity, regions, funding, number of citations, and influencing factors were summarized as the main elements of adherence to the reporting quality with quantitative results shown in Fig 3A.

One important quality indicator for sufficient study design was the reporting of preservation techniques and treatment. Moreover, following the ARRIVE guidelines to enhance the quality and replicability of the study should also result in a heightened positive impact on animal welfare. This is due to the inclusion of procedure particulars and ethical declarations, providing comprehensive information. Surprisingly, despite the fact that only two of these publications was conducted before 2010, the use of postoperative analgesia (n = 46, 82.1%) and antibiotics (n = 46, 82.1%) was not reported in the majority of studies. According to the general principles of animal research, experimental surgery as well as good veterinarian practice, the administration of postoperative analgesia for a minimum of 48 hours after surgery is mandatory and shall be tracked by continuous follow-up on evaluation for signs of pain [36]. Although the ARRIVE checklist does not explicitly mention the documentation of analgesic treatment, it is still necessary to include it under Item 7a, as a comprehensive description of all experimental procedures and an essential component of a proper reporting on animal experiments. Strikingly, based on our results, the reporting of adverse events and the evaluation of the 3Rs (replacement, reduction, and refinement) [37] are among the least frequently listed information.

Since their introduction in 2010, the ARRIVE guidelines have been endorsed by approximately 600 journals. However, we were not able to find a significant improvement in this very specialized topic independent of endorsement of the ARRIVE guidelines or significant differences within the pre- and post-ARRIVE era. Our findings are in line with other systematic reviews focusing on the implementation of ARRIVE guidelines in various clinical models [38–42]. When assessing ARRIVE scores in both the pre- and post-ARRIVE periods, it’s important to acknowledge that the field of mechanical perfusion is relatively new, which could introduce bias into the results. This bias may stem from the limited number of publications available from the pre-ARRIVE era.

Sufficient funding is of utmost importance in all stages of research, and also should be considered during the planning of animal studies [43]. Information on all funding resources was one of the most increased items in the post-ARRIVE evaluation. This is especially true when it comes to demanding animal models like LT, which requires a significant amount of manpower and resources. However, characteristics in funding did not have a direct effect to reporting quality. While it is important to adhere to reporting guidelines for the ethical treatment of animals in research, it is worth noting that the quality of reporting does not necessarily reflect the quality of the research itself.

## Limitations

Even though our study comprises a unique dataset of preclinical studies using machine perfusion in the setting of *in vivo* LT models and describes important characteristics, it has various limitations. The considerable heterogeneity often observed in animal studies limits the results of this systematic review to a primarily descriptive analysis. Due to our prior experience with preclinical systematic reviews that involved studies with high heterogeneity, we did not aim to conduct a meta-analyses of study outcomes or quantitative data synthesis.

We only included studies in English language which excluded 29 studies during the abstract-title screening, thus, these could not contribute to this systematic review. Another limitation is the selection of models restricted to machine perfusion solely. This very specialized field is of high interest for human medicine but young. Therefore, we only had 13 studies (23.2%) contributing to the pre-ARRIVE era. We also did not include conference paper as these are mostly not peer-reviewed. Therefore, we were not able to include the most recent studies.

## Conclusion

In summary, our systematic review assessed adherence to the ARRIVE guidelines regarding the use of animal models in machine perfusion for liver transplantation. Our findings indicate that there is still heterogeneity in publication and reporting quality and a lack of recognition of these guidelines on a global scale. In 2014, Baker et al. reported that following all aspects of the ARRIVE guidelines can be difficult under the current reporting norms in biomedical science since the guidelines are not yet integrated into editorial norms. Unless there are major changes in editorial policies, it is unlikely that the guidelines will be implemented in all stages. However, like our group almost a decade later, Baker et al. also found that certain reporting standards, such as reporting on ethical approval obtained before publication, have been successfully implemented and can influence reporting behavior [44]. Since then, a large number of publishers and journals have adopted the ARRIVE checklist into their submission guidelines. Nevertheless, complete compliance with the ARRIVE criteria remains lacking, and improvement is not guaranteed in all aspects. Particularly in topics related to animal welfare, such as analgesics and other welfare-related treatments as well as the principles of the 3Rs (replacement, reduction, and refinement) our study highlights the need for further improvement. By adhering to comprehensive reporting standards like the ARRIVE guidelines, we can ensure that research is conducted in a way that is both scientifically rigorous and ethically responsible.

## Supporting information

S1 Table Details of the ARRIVE score grading system. (DOCX)

S2 Table Complete list of supporting references. (DOCX)

S3 Table Full search strategy. (DOCX)

S1 Fig Cooperation network in multinational studies. The scale of the cluster represented the articles’ number of corresponding country and the size variation of the line between countries represented the cooperation intensity. Connections of ≥1 were visualized.
(TIF)

S1 File Original study description. (XLSX)

S2 File Prisma abstract checklist. (DOCX)

S3 File Prisma checklist. (DOCX)

S4 File Study protocol. (PDF)

## Abbreviation

ARRIVE: Animal Research: Reporting of *In Vivo* Experiments
CONSORT: Consolidated Standards of Reporting Trials
HMP: hypothermic machine perfusion
HNPE: hypothermic nitrogen machine perfusion
HOPE: hypothermic oxygenated machine perfusion
LT: liver transplantation
MP: machine perfusion
NC3Rs: National Centre for the Replacement and Reduction of Animal Experiments
NMP: normothermic machine perfusion
RCT: randomized controlled trials
SNMP: subnormothermic machine perfusion
