# Supplementary material for: Machine perfusion of the liver and in vivo animal models: A systematic review of the preclinical research landscape
Source: PLoS One. 2024 Feb 8;19(2):e0297942. doi: 10.1371/journal.pone.0297942 (PMC10852327; doi:10.1371/journal.pone.0297942)
Supplement: S1 Table — (DOCX) [file pone.0297942.s001.docx]

| Item | | | Description | Number | Total score  *(adapted from Schwarz et al. (1))* |
| --- | --- | --- | --- | --- | --- |
| TITLE | *Provide as accurate and concise a description of the content of the article as possible.* | | | 1 | 0 = clearly insufficient  1 = possibly sufficient  2 = clearly sufficient |
| ABSTRACT | *Provide an accurate summary of the background, research objectives (including details of the species or strain of animal used), key methods, principal findings, and conclusions of the study.* | | | 2 | 0 = clearly insufficient  1 = possibly sufficient  2 = clearly sufficient |
| INTRODUCTION | | | |  |  |
| Background | | *Include sufficient scientific background (including relevant references to previous work) to understand the motivation and context for the study, and explain the experimental approach and rationale* | | 3a | 0 = clearly insufficient  1 = possibly sufficient  2 = clearly sufficient |
|  | | *Explain how and why the animal species and model being used can address the scientific objectives and, where appropriate, the study’s relevance to human biology* | | 3b | 0 = clearly insufficient  1 = possibly sufficient  2 = clearly sufficient |
| Objectives | | *Clearly describe the primary and any secondary objectives of the study, or specific hypotheses being tested)* | | 4 | 0=not clear  2=clear |
| METHODS | |  | |  |  |
| Ethical statement | | *Indicate the nature of the ethical review permissions, relevant licences (e.g. Animal [Scientific Procedures] Act 1986), and national or institutional guidelines for the care and use of animals, that cover the research* | | 5 | 0=no  2=yes |
| Study design | | *For each experiment, give brief details of the study design, including:*  *Number of experimental*  *and control groups.* | | 6a | 0=no/not clear  2=clear |
|  |  | *Any steps taken to minimise the effects of subjective bias when allocating animals to treatment (e.g., randomisation procedure) and when assessing results (e.g., if done, describe who was blinded and when).* | | 6b | 0 = clearly insufficient  1 = possibly sufficient  2 = clearly sufficient |
|  |  | *The experimental unit (e.g. a single animal, group, or cage of animals).* | | 6c | 0=no  2=yes |
|  |  | *A time-line diagram or flow chart can be useful to illustrate how complex study designs were carried out.* | | 6d | 0=no  2=yes |
| Experimental procedures | | *For each experiment and each experimental group, including controls, provide precise details of all procedures carried out.*  *How (e.g. drug formulation and dose, site and route of administration, anaesthesia and analgesia used [including monitoring], surgical (procedure, method of euthanasia). Provide details of any specialist equipment used, including supplier(s).* | | 7a | 0 = clearly insufficient  1 = possibly sufficient  2 = clearly sufficient |
|  |  | *Where (e.g. home cage, laboratory, water maze )* | | 7b | 0=not clear  2= clear |
|  |  | *When (e.g. time. e of day)* | | 7c | 0=not clear  2= clear |
|  |  | *Why (e.g. rationale for choice of specific anaesthetic, route of administration, drug dose used)* | | 7d | 0 = clearly insufficient  1 = possibly sufficient  2 = clearly sufficient |
| Experimental animals | | *Provide details of the animals used, including species, strain, sex, developmental stage (e.g., mean or median age plus age range), and weight (e.g., mean or median weight plus weight range).* | | 8a | 0= <3 criteria  1= 3-4 criteria  2= 5 criteria |
|  |  | *Provide further relevant information such as the source of animals, international strain nomenclature, genetic modification status (e.g. knock-out or transgenic), genotype, health/immune status, drug- or- test naive, previous procedures, etc.* | | 8b | 0 = clearly insufficient  1 = possibly sufficient  2 = clearly sufficient |
| Housing and husbandry | | *Provide details of:*  *Housing (e.g., type of facility, e.g., specific pathogen free (SPF); type of cage or housing; bedding*  *material; number of cage companions).* | | 9a | <1 criteria=0  1-3 criteria=1  4 criteria=2 |
|  |  | *Husbandry conditions (e.g., breeding programme, light/dark cycle, temperature, type of food, access to food and water, environmental enrichment).* | | 9b | <2 criteria=0  2-4 criteria=1  5 criteria=2 |
|  |  | *Welfare-related assessments and interventions that were carried out before, during, or after the*  *experiment.* | | 9c | 0 = clearly insufficient  1 = possibly sufficient  2 = clearly sufficient |
| Sample size | | *Specify the total number of animals used in each experiment and the number of animals in each experimental group* | | 10a | 0= not clear  2=clear |
|  |  | *Explain how the number of animals was decided. Provide details of any sample size calculation used.* | | 10b | 0=no  2=yes |
|  |  | *Indicate the number of independent replications of each experiment, if relevant.* | | 10c | 0=no  2=yes |
| Allocating animals to  experimental groups | | *Give full details of how animals were allocated to experimental groups, including randomisation or*  *matching if done* | | 11a | 0=no  2=yes |
|  |  | *Describe the order in which the animals in the different experimental groups were treated and assessed.* | | 11b | 0=no  2=yes |
| Experimental outcomes | | *Clearly define the primary and secondary experimental outcomes assessed* | | 12 | 0= not clear  2=clear |
| Statistical methods | | *Provide details of the statistical methods used for each analysis.* | | 13a | 0= no  2= yes |
|  |  | *Specify the unit of analysis for each dataset (e.g. single animal, group of animals, single neuron).* | | 13b | 0=no  2=yes |
|  |  | *Describe any methods used to assess whether the data met the assumptions of the statistical*  *approach.* | | 13c | 0=no  2=yes |
| Results | |  | |  |  |
| Baseline data | | *Characteristics and health status of animals* | | 14 | 0 = clearly insufficient  1 = possibly sufficient  2 = clearly sufficient |
| Numbers analysed | | *Report the number of animals in each group included in each analysis. Report absolute numbers (e.g. 10/20, not 50%2* | | 15a | 0= no  2=yes |
|  | | *If any animals or data were not included in the analysis, explain why.* | | 15b | 0=no  2=yes |
| Outcomes and  Estimation | | *Report the results for each analysis carried out, with a measure of precision (e.g. standard error or confidence interval).* | | 16 | 0= no  1= partially  2= yes |
| Adverse events | | *Give details of all important adverse events in each experimental group.* | | 17a | 0 = clearly insufficient  1 = possibly sufficient  2 = clearly sufficient |
|  |  | *Describe any modifications to the experimental protocols made to reduce adverse events.* | | 17b | 0= not clear  2=clear |
| Discussion | |  | |  |  |
| Interpretation/scientific  implications | | *Interpret the results, taking into account the study objectives and hypotheses, current theory and other relevant studies in the literature.* | | 18a | 0 = clearly insufficient  1 = possibly sufficient  2 = clearly sufficient |
|  |  | *Comment on the study limitations including any potential sources of bias, any limitations of the animal model, and the imprecision associated with the results* | | 18b | 0 = clearly insufficient  1 = possibly sufficient  2 = clearly sufficient |
|  |  | *Describe any implications of your experimental methods or findings for the*  *replacement, refinement or reduction (the 3Rs) of the use of animals in research* | | 18c | 0= no  2= yes |
| Generalisability/translation | | *Comment on whether, and how, the findings of this study are likely to translate to other species or systems, including any relevance to human biology.* | | 19 | 0 = clearly insufficient  1 = possibly sufficient  2 = clearly sufficient |
| Funding | | *List all funding sources (including grant number) and the role of the funder(s) in the study.* | | 20 | 0= no  2=yes |

**Supplementary Table References:**

1. Schwarz F, Iglhaut G, Becker J. Quality assessment of reporting of animal studies on pathogenesis and treatment of peri-implant mucositis and peri-implantitis. A systematic review using the ARRIVE guidelines. Journal of clinical periodontology. 2012;39 Suppl 12:63-72.
