# Supplementary material for: Machine perfusion of the liver and in vivo animal models: A systematic review of the preclinical research landscape
Source: PLoS One. 2024 Feb 8;19(2):e0297942. doi: 10.1371/journal.pone.0297942 (PMC10852327; doi:10.1371/journal.pone.0297942)
Supplement: S2 Table — (DOCX) [file pone.0297942.s002.docx]

**S2 File: Complete list of supporting references**

Supplementary material for: W.J. Liu et al. 3 Decades of pre-clinical Ex vivo machine perfusion in liver transplantation: A systematic review on publication quality

1. Shigeta T, Matsuno N, Huai-Che H, Obara H, Mizunuma H, Hirano T, Uemoto S, Enosawa S. A basic consideration for porcine liver preservation using a novel continuous machine perfusion device. Transplant Proc. 2012 May;44(4):942-5. doi: 10.1016/j.transproceed.2012.03.013. PMID: 22564591.
2. Gringeri E, Polacco M, D'Amico FE, Scopelliti M, Bassi D, Bonsignore P, Luisetto R, Lodo E, Carraro A, Zanus G, Cillo U. A new liver autotransplantation technique using subnormothermic machine perfusion for organ preservation in a porcine model. Transplant Proc. 2011 May;43(4):997-1000. doi: 10.1016/j.transproceed.2011.01.139. PMID: 21620035.
3. Berendsen TA, Bruinsma BG, Lee J, D'Andrea V, Liu Q, Izamis ML, Uygun K, Yarmush ML. A simplified subnormothermic machine perfusion system restores ischemically damaged liver grafts in a rat model of orthotopic liver transplantation. Transplant Res. 2012 May 9;1(1):6. doi: 10.1186/2047-1440-1-6. PMID: 23369351; PMCID: PMC3552573.
4. Alix P, Val-Laillet D, Turlin B, Ben Mosbah I, Burel A, Bobillier E, Bendavid C, Delpy E, Zal F, Corlu A, Boudjema K. Adding the oxygen carrier M101 to a cold-storage solution could be an alternative to HOPE for liver graft preservation. JHEP Rep. 2020 May 8;2(4):100119. doi: 10.1016/j.jhepr.2020.100119. PMID: 32695967; PMCID: PMC7364164.
5. Compagnon P, Levesque E, Hentati H, Disabato M, Calderaro J, Feray C, Corlu A, Cohen JL, Ben Mosbah I, Azoulay D. An Oxygenated and Transportable Machine Perfusion System Fully Rescues Liver Grafts Exposed to Lethal Ischemic Damage in a Pig Model of DCD Liver Transplantation. Transplantation. 2017 Jul;101(7):e205-e213. doi: 10.1097/TP.0000000000001764. PMID: 28403128.
6. Goldaracena N, Echeverri J, Spetzler VN, Kaths JM, Barbas AS, Louis KS, Adeyi OA, Grant DR, Selzner N, Selzner M. Anti-inflammatory signaling during ex vivo liver perfusion improves the preservation of pig liver grafts before transplantation. Liver Transpl. 2016 Nov;22(11):1573-1583. doi: 10.1002/lt.24603. PMID: 27556578.
7. Iwamoto H, Matsuno N, Narumi Y, Uchiyama M, Kozaki K, Degawa H, Hama K, Kikuchi K, Takeuchi H, Kozaki M, Nagao T. Beneficial effect of machine perfusion preservation on liver transplantation from non-heart-beating donors. Transplant Proc. 2000 Nov;32(7):1645-6. doi: 10.1016/s0041-1345(00)01437-8. PMID: 11119873.
8. Wu L, Cao H, Tian X, Zheng W, Yuan M, Li X, Tian X, Wang Y, Song H. Bone marrow mesenchymal stem cells modified with heme oxygenase-1 alleviate rejection of donation after circulatory death liver transplantation by inhibiting dendritic cell maturation in rats. Int Immunopharmacol. 2022 Jun;107:108643. doi: 10.1016/j.intimp.2022.108643. Epub 2022 Feb 28. PMID: 35240383.
9. Uchiyama M, Matsuno N, Hama K, Iwamoto H, Narumi Y, Kikuchi K, Degawa H, Kozaki K, Kubota K, Takeuchi H, Sakurai E, Asuwa N, Masuda Toshihiko Hirano S, Nagao T. Comparison between nonpulsatile and pulsatile machine perfusion preservation in liver transplantation from non-heart-beating donors. Transplant Proc. 2001 Feb-Mar;33(1-2):936-8. doi: 10.1016/s0041-1345(00)02276-4. PMID: 11267136.
10. Echeverri J, Goldaracena N, Kaths JM, Linares I, Roizales R, Kollmann D, Hamar M, Urbanellis P, Ganesh S, Adeyi OA, Tazari M, Selzner M, Selzner N. Comparison of BQ123, Epoprostenol, and Verapamil as Vasodilators During Normothermic Ex Vivo Liver Machine Perfusion. Transplantation. 2018 Apr;102(4):601-608. doi: 10.1097/TP.0000000000002021. PMID: 29189484.
11. Yoshimoto S, Torai S, Yoshioka M, Nadahara S, Kobayashi E. Continuous Resuscitation for Porcine Liver Transplantation From Donor After Cardiac Death. Transplant Proc. 2019 Jun;51(5):1463-1467. doi: 10.1016/j.transproceed.2019.03.016. PMID: 31155180.
12. Bonaccorsi-Riani E, Gillooly AR, Iesari S, Brüggenwirth IMA, Ferguson CM, Komuta M, Xhema D, Daumerie A, Maistriaux L, Leuvenink H, Kupiec-Weglinski J, Porte RJ, Khvorova A, Cave DR, Gianello P, Martins PN. Delivering siRNA Compounds During HOPE to Modulate Organ Function: A Proof-of-concept Study in a Rat Liver Transplant Model. Transplantation. 2022 Aug 1;106(8):1565-1576. doi: 10.1097/TP.0000000000004175. Epub 2022 May 18. PMID: 35581683.
13. Bruinsma BG, Berendsen TA, Izamis ML, Yarmush ML, Uygun K. Determination and extension of the limits to static cold storage using subnormothermic machine perfusion. Int J Artif Organs. 2013 Nov;36(11):775-80. doi: 10.5301/ijao.5000250. Epub 2013 Oct 2. PMID: 24338652; PMCID: PMC4091033.
14. Yu Y, Cheng Y, Pan Q, Zhang YJ, Jia DG, Liu YF. Effect of the Selective NLRP3 Inflammasome Inhibitor mcc950 on Transplantation Outcome in a Pig Liver Transplantation Model With Organs From Donors After Circulatory Death Preserved by Hypothermic Machine Perfusion. Transplantation. 2019 Feb;103(2):353-362. doi: 10.1097/TP.0000000000002461. PMID: 30247318.
15. Zhang Y, Pan Q, Cheng Y, Liu Y. Effects of SP600125 and hypothermic machine perfusion on livers donated after cardiac death in a pig allograft transplantation model. Eur J Med Res. 2021 Feb 5;26(1):15. doi: 10.1186/s40001-020-00472-9. PMID: 33546770; PMCID: PMC7863371.
16. Zhao DF, Dong Q, Zhang T. Effects of Static Cold Storage and Hypothermic Machine Perfusion on Oxidative Stress Factors, Adhesion Molecules, and Zinc Finger Transcription Factor Proteins Before and After Liver Transplantation. Ann Transplant. 2017 Feb 17;22:96-100. doi: 10.12659/aot.901897. PMID: 28209945.
17. Shigeta T, Matsuno N, Obara H, Mizunuma H, Kanazawa H, Tanaka H, Fukuda A, Sakamoto S, Kasahara M, Uemoto S, Enosawa S. Functional recovery of donation after cardiac death liver graft by continuous machine perfusion preservation in pigs. Transplant Proc. 2012 May;44(4):946-7. doi: 10.1016/j.transproceed.2012.01.078. PMID: 22564592.
18. Jia JJ, Xie HY, Li JH, He Y, Jiang L, He N, Zhou L, Wang W, Zheng SS. Graft protection of the liver by hypothermic machine perfusion involves recovery of graft regeneration in rats. J Int Med Res. 2019 Jan;47(1):427-437. doi: 10.1177/0300060518787726. PMID: 30791830; PMCID: PMC6384453.
19. Cao H, Wu L, Tian X, Zheng W, Yuan M, Li X, Tian X, Wang Y, Song H, Shen Z. HO-1/BMMSC perfusion using a normothermic machine perfusion system reduces the acute rejection of DCD liver transplantation by regulating NKT cell co-inhibitory receptors in rats. Stem Cell Res Ther. 2021 Nov 24;12(1):587. doi: 10.1186/s13287-021-02647-5. PMID: 34819139; PMCID: PMC8611848.
20. Vekemans K, Liu Q, Heedfeld V, Van de Vel K, Wylin T, Pirenne J, Monbaliu D. Hypothermic Liver Machine Perfusion With EKPS-1 Solution vs Aqix RS-I Solution: In Vivo Feasibility Study in a Pig Transplantation Model. Transplant Proc. 2009 Mar;41(2):617-21. doi: 10.1016/j.transproceed.2008.12.022. PMID: 19328939.
21. Guarrera JV, Estevez J, Boykin J, Boyce R, Rashid J, Sun S, Arrington B. Hypothermic machine perfusion of liver grafts for transplantation: technical development in human discard and miniature swine models. Transplant Proc. 2005 Jan-Feb;37(1):323-5. doi: 10.1016/j.transproceed.2004.12.094. PMID: 15808631.
22. Monbaliu D, Heedfeld V, Liu Q, Wylin T, van Pelt J, Vekemans K, Pirenne J. Hypothermic machine perfusion of the liver: is it more complex than for the kidney? Transplant Proc. 2011 Nov;43(9):3445-50. doi: 10.1016/j.transproceed.2011.09.033. PMID: 22099817.
23. Fondevila C, Hessheimer AJ, Maathuis MH, Muñoz J, Taurá P, Calatayud D, Leuvenink H, Rimola A, García-Valdecasas JC, Ploeg RJ. Hypothermic oxygenated machine perfusion in porcine donation after circulatory determination of death liver transplant. Transplantation. 2012 Jul 15;94(1):22-9. doi: 10.1097/TP.0b013e31825774d7. PMID: 22691959.
24. Schlegel A, Kron P, Graf R, Clavien PA, Dutkowski P. Hypothermic Oxygenated Perfusion (HOPE) downregulates the immune response in a rat model of liver transplantation. Ann Surg. 2014 Nov;260(5):931-7; discussion 937-8. doi: 10.1097/SLA.0000000000000941. PMID: 25243553.
25. Kron P, Schlegel A, Mancina L, Clavien PA, Dutkowski P. Hypothermic oxygenated perfusion (HOPE) for fatty liver grafts in rats and humans. J Hepatol. 2017 Sep 21:S0168-8278(17)32268-7. doi: 10.1016/j.jhep.2017.08.028. Epub ahead of print. PMID: 28870676.
26. Schlegel A, Graf R, Clavien PA, Dutkowski P. Hypothermic oxygenated perfusion (HOPE) protects from biliary injury in a rodent model of DCD liver transplantation. J Hepatol. 2013 Nov;59(5):984-91. doi: 10.1016/j.jhep.2013.06.022. Epub 2013 Jun 29. PMID: 23820408.
27. Schlegel A, Muller X, Mueller M, Stepanova A, Kron P, de Rougemont O, Muiesan P, Clavien PA, Galkin A, Meierhofer D, Dutkowski P. Hypothermic oxygenated perfusion protects from mitochondrial injury before liver transplantation. EBioMedicine. 2020 Oct;60:103014. doi: 10.1016/j.ebiom.2020.103014. Epub 2020 Sep 24. PMID: 32979838; PMCID: PMC7519249.
28. Lin F, Zhen F, Yan X, Shaojun Y, Guizhu P, Yanfeng W, Qifa Y. Hypothermic oxygenated perfusion with defatting cocktail further improves steatotic liver grafts in a transplantation rat model. Artif Organs. 2021 Sep;45(9):E304-E316. doi: 10.1111/aor.13976. Epub 2021 Jun 9. PMID: 33908066.
29. Linares-Cervantes I, Kollmann D, Goto T, Echeverri J, Kaths JM, Hamar M, Urbanellis P, Mazilescu L, Rosales R, Bruguera C, Oquendo F, Ganesh S, Adeyi OA, Yip P, Selzner N, Selzner M. Impact of Different Clinical Perfusates During Normothermic Ex Situ Liver Perfusion on Pig Liver Transplant Outcomes in a DCD Model. Transplant Direct. 2019 Mar 4;5(4):e437. doi: 10.1097/TXD.0000000000000876. PMID: 30993191; PMCID: PMC6445654.
30. Shigeta T, Matsuno N, Obara H, Kanazawa H, Tanaka H, Fukuda A, Sakamoto S, Kasahara M, Mizunuma H, Enosawa S. Impact of rewarming preservation by continuous machine perfusion: improved post-transplant recovery in pigs. Transplant Proc. 2013 Jun;45(5):1684-9. doi: 10.1016/j.transproceed.2013.01.098. PMID: 23769024.
31. Jia D, Pan Q, Zhang Y, Yu Y, Song Z, Liu YF, Jia Z, Guo S, Cheng Y. Ischemic postconditioning improves the outcome of organs from donors after cardiac death in a pig liver transplantation model and provides synergistic protection with hypothermic machine perfusion. Clin Transplant. 2021 Oct;35(10):e14417. doi: 10.1111/ctr.14417. Epub 2021 Jul 11. PMID: 34231926.
32. Fontes P, Lopez R, van der Plaats A, Vodovotz Y, Minervini M, Scott V, Soltys K, Shiva S, Paranjpe S, Sadowsky D, Barclay D, Zamora R, Stolz D, Demetris A, Michalopoulos G, Marsh JW. Liver preservation with machine perfusion and a newly developed cell-free oxygen carrier solution under subnormothermic conditions. Am J Transplant. 2015 Feb;15(2):381-94. doi: 10.1111/ajt.12991. PMID: 25612645; PMCID: PMC5024042.
33. Uchiyama M, Kozaki K, Nemoto T, Degawa H, Matsuno N, Kubota K, Takeuchi H, Sakurai E, Kozaki M, Ikeda T, Asuwa N, Masuda S, Nagao T. Liver transplantation from non-heart-beating donors: effect of machine perfusion preservation and pentoxifylline. Transplant Proc. 1998 Nov;30(7):3798-800. doi: 10.1016/s0041-1345(98)01242-1. PMID: 9838665.
34. Sadowsky D, Zamora R, Barclay D, Yin J, Fontes P, Vodovotz Y. Machine Perfusion of Porcine Livers with Oxygen-Carrying Solution Results in Reprogramming of Dynamic Inflammation Networks. Front Pharmacol. 2016 Nov 4;7:413. doi: 10.3389/fphar.2016.00413. PMID: 27867357; PMCID: PMC5095594.
35. Matsuno N, Uchiyama M, Iwamoto H, Hama K, Narumi Y, Kikuchi K, Degawa H, Kozaki K, Nagao T. Machine perfusion preservation for liver transplantation from non-heart-beating donors with agonal stage. Transplant Proc. 2002 Nov;34(7):2610-1. doi: 10.1016/s0041-1345(02)03444-9. PMID: 12431543.
36. Yagi S, Ito T, Shirai H, Yao S, Masano Y, Ogawa E, Gabata R, Uemoto S, Kobayashi E. Micro- and macro-borderless surgery using a newly developed high-resolution (4K) three-dimensional video system. PLoS One. 2021 May 12;16(5):e0250559. doi: 10.1371/journal.pone.0250559. PMID: 33979347; PMCID: PMC8115828.
37. Dutkowski P, Furrer K, Tian Y, Graf R, Clavien PA. Novel short-term hypothermic oxygenated perfusion (HOPE) system prevents injury in rat liver graft from non-heart beating donor. Ann Surg. 2006 Dec;244(6):968-76; discussion 976-7. doi: 10.1097/01.sla.0000247056.85590.6b. PMID: 17122622; PMCID: PMC1856639.
38. de Rougemont O, Breitenstein S, Leskosek B, Weber A, Graf R, Clavien PA, Dutkowski P. One hour hypothermic oxygenated perfusion (HOPE) protects nonviable liver allografts donated after cardiac death. Ann Surg. 2009 Nov;250(5):674-83. doi: 10.1097/SLA.0b013e3181bcb1ee. PMID: 19806056.
39. He N, Jia JJ, Xie HY, Li JH, He Y, Yin SY, Liang RP, Jiang L, Liu JF, Xu KD, Zhang ZH, Zhou L, Zheng SS. Partial Inhibition of HO-1 Attenuates HMP-Induced Hepatic Regeneration against Liver Injury in Rats. Oxid Med Cell Longev. 2018 Apr 15;2018:9108483. doi: 10.1155/2018/9108483. PMID: 29849924; PMCID: PMC5925174.
40. Linares-Cervantes I, Echeverri J, Cleland S, Kaths JM, Rosales R, Goto T, Kollmann D, Hamar M, Urbanellis P, Mazilescu L, Ganesh S, Adeyi OA, Yip P, Goryńska P, Bojko B, Goryński K, Grant DR, Selzner N, Wąsowicz M, Selzner M. Predictor parameters of liver viability during porcine normothermic ex situ liver perfusion in a model of liver transplantation with marginal grafts. Am J Transplant. 2019 Nov;19(11):2991-3005. doi: 10.1111/ajt.15395. Epub 2019 May 28. PMID: 31012532.
41. Yoshimoto S, Soyama A, Fukumoto M, Hara T, Hidaka M, Torai S, Kasamatsu H, Ishikawa J, Ohara M, Kobayashi E, Eguchi S. Preliminary Observations of An Ex Vivo Normothermic Whole Blood Machine Perfusion in An Experimental Liver Transplant Porcine Model. Transplant Proc. 2023 May;55(4):1005-1011. doi: 10.1016/j.transproceed.2023.03.067. Epub 2023 Apr 26. PMID: 37117106.
42. Tolboom H, Pouw RE, Izamis ML, Milwid JM, Sharma N, Soto-Gutierrez A, Nahmias Y, Uygun K, Berthiaume F, Yarmush ML. Recovery of warm ischemic rat liver grafts by normothermic extracorporeal perfusion. Transplantation. 2009 Jan 27;87(2):170-7. doi: 10.1097/TP.0b013e318192df6b. PMID: 19155970; PMCID: PMC2743395.
43. Izamis ML, Tolboom H, Uygun B, Berthiaume F, Yarmush ML, Uygun K. Resuscitation of ischemic donor livers with normothermic machine perfusion: a metabolic flux analysis of treatment in rats. PLoS One. 2013 Jul 26;8(7):e69758. doi: 10.1371/journal.pone.0069758. PMID: 23922793; PMCID: PMC3724866.
44. Matsuno N, Obara H, Watanabe R, Iwata S, Kono S, Fujiyama M, Hirano T, Kanazawa H, Enosawa S. Rewarming preservation by organ perfusion system for donation after cardiac death liver grafts in pigs. Transplant Proc. 2014 May;46(4):1095-8. doi: 10.1016/j.transproceed.2013.12.035. PMID: 24815137.
45. Pienaar BH, Lindell SL, Van Gulik T, Southard JH, Belzer FO. Seventy-two-hour preservation of the canine liver by machine perfusion. Transplantation. 1990 Feb;49(2):258-60. doi: 10.1097/00007890-199002000-00005. PMID: 2305453.
46. Tolboom H, Izamis ML, Sharma N, Milwid JM, Uygun B, Berthiaume F, Uygun K, Yarmush ML. Subnormothermic machine perfusion at both 20°C and 30°C recovers ischemic rat livers for successful transplantation. J Surg Res. 2012 Jun 1;175(1):149-56. doi: 10.1016/j.jss.2011.03.003. Epub 2011 Mar 29. PMID: 21550058; PMCID: PMC3863393.
47. Vogel T, Brockmann JG, Pigott D, Neil DAH, Muthusamy ASR, Coussios CC, Friend PJ. Successful transplantation of porcine liver grafts following 48-hour normothermic preservation. PLoS One. 2017 Nov 27;12(11):e0188494. doi: 10.1371/journal.pone.0188494. PMID: 29176869; PMCID: PMC5703476.
48. Bruinsma BG, Berendsen TA, Izamis ML, Yeh H, Yarmush ML, Uygun K. Supercooling preservation and transplantation of the rat liver. Nat Protoc. 2015 Mar;10(3):484-94. doi: 10.1038/nprot.2015.011. Epub 2015 Feb 18. PMID: 25692985; PMCID: PMC4494653.
49. Fondevila C, Hessheimer AJ, Maathuis MH, Muñoz J, Taurá P, Calatayud D, Leuvenink H, Rimola A, Ploeg RJ, García-Valdecasas JC. Superior preservation of DCD livers with continuous normothermic perfusion. Ann Surg. 2011 Dec;254(6):1000-7. doi: 10.1097/SLA.0b013e31822b8b2f. PMID: 21862925.
50. Lee CY, Jain S, Duncan HM, Zhang JX, Jones JW Jr, Southard JH, Clemens MG. Survival transplantation of preserved non-heart-beating donor rat livers: preservation by hypothermic machine perfusion. Transplantation. 2003 Nov 27;76(10):1432-6. doi: 10.1097/01.TP.0000088674.23805.0F. PMID: 14657681.
51. Oldani G, Peloso A, Slits F, Gex Q, Delaune V, Orci LA, van de Looij Y, Colin DJ, Germain S, de Vito C, Rubbia-Brandt L, Lacotte S, Toso C. The impact of short-term machine perfusion on the risk of cancer recurrence after rat liver transplantation with donors after circulatory death. PLoS One. 2019 Nov 25;14(11):e0224890. doi: 10.1371/journal.pone.0224890. PMID: 31765399; PMCID: PMC6876876.
52. Carnevale ME, Lausada N, Juan de Paz L, Stringa P, Machuca M, Rumbo M, Guibert EE, Tiribelli C, Gondolesi GE, Rodriguez JV. The Novel N,N-bis-2-Hydroxyethyl-2-Aminoethanesulfonic Acid-Gluconate-Polyethylene Glycol-Hypothermic Machine Perfusion Solution Improves Static Cold Storage and Reduces Ischemia/Reperfusion Injury in Rat Liver Transplant. Liver Transpl. 2019 Sep;25(9):1375-1386. doi: 10.1002/lt.25573. Epub 2019 Jul 4. PMID: 31121085.
53. Abraham N, Zhang M, Cray P, Gao Q, Samy KP, Neill R, Cywinska G, Migaly J, Kahan R, Pontula A, Halpern SE, Rush C, Penaflor J, Kesseli SJ, Krischak M, Song M, Hartwig MG, Pollara JJ, Barbas AS. Two Compartment Evaluation of Liver Grafts During Acellular Room Temperature Machine Perfusion (acRTMP) in a Rat Liver Transplant Model. Front Med (Lausanne). 2022 Feb 24;9:804834. doi: 10.3389/fmed.2022.804834. PMID: 35280912; PMCID: PMC8907827.
54. Kozaki K, Uchiyama M, Nemoto T, Degawa H, Takeuchi H, Matsuno N, Sakurai E, Kozaki M, Nagao T. Usefulness of a combination of machine perfusion and pentoxifylline for porcine liver transplantation from non-heart-beating donors with prolonged hypotension. Transplant Proc. 1997 Dec;29(8):3476-7. doi: 10.1016/s0041-1345(97)00986-x. PMID: 9414799.
55. Uchiyama M, Matsuno N, Nakamura Y, Iwamoto H, Hama K, Narumi K, Kikuchi K, Kubota K, Takeuchi H, Sakurai E, Nagao T. Usefulness of preservation by machine perfusion of liver grafts from non-heart-beating donors-a porcine model. Transplant Proc. 2003 Feb;35(1):105-6. doi: 10.1016/s0041-1345(02)03801-0. PMID: 12591327.
56. Schlegel A, Kron P, Graf R, Dutkowski P, Clavien PA. Warm vs. cold perfusion techniques to rescue rodent liver grafts. J Hepatol. 2014 Dec;61(6):1267-75. doi: 10.1016/j.jhep.2014.07.023. Epub 2014 Jul 31. PMID: 25086285.
