# Supplementary material for: Machine perfusion of the liver and in vivo animal models: A systematic review of the preclinical research landscape
Source: PLoS One. 2024 Feb 8;19(2):e0297942. doi: 10.1371/journal.pone.0297942 (PMC10852327; doi:10.1371/journal.pone.0297942)
Supplement: S3 Table — (DOCX) [file pone.0297942.s003.docx]

**Full search strategy: 09.08.2023**

**Database: Pubmed**

Liver[Title/Abstract] OR hepatic[Title/Abstract] OR "liver transplantation"[Title/Abstract] OR "hepatic transplantation"[Title/Abstract] OR "liver transplant"[Title/Abstract] OR "hepatic transplant"[Title/Abstract] AND "machine perfusion"[Title/Abstract] OR "machine preservation"[Title/Abstract]

<https://pubmed.ncbi.nlm.nih.gov/?term=Liver%5BTitle%2FAbstract%5D+OR+hepatic%5BTitle%2FAbstract%5D+OR+%22liver+transplantation%22%5BTitle%2FAbstract%5D+OR+%22hepatic+transplantation%22%5BTitle%2FAbstract%5D+OR+%22liver+transplant%22%5BTitle%2FAbstract%5D+OR+%22hepatic+transplant%22%5BTitle%2FAbstract%5D+AND+%22machine+perfusion%22%5BTitle%2FAbstract%5D+OR+%22machine+preservation%22%5BTitle%2FAbstract%5D>

**Database: Embase**

(liver:ti,ab,kw OR hepatic:ti,ab,kw OR 'liver transplantation':ti,ab,kw OR 'hepatic transplantation':ti,ab,kw OR 'liver transplant':ti,ab,kw OR 'hepatic transplant':ti,ab,kw) AND ('machine perfusion':ti,ab,kw OR 'machine preservation':ti,ab,kw)

**Database: Web of Science**

(liver OR hepatic OR "liver transplantation" OR "hepatic transplantation" OR "liver transplant" OR "hepatic transplant") (Topic) AND ("machine perfusion" OR "machine preservation") (Topic)

https://www.webofscience.com/wos/woscc/summary/a230d307-be40-4dec-b627-66cda3c43ae4-ac9e621b/relevance/1
