# Supplementary figures and images for: Machine perfusion of the liver and in vivo animal models: A systematic review of the preclinical research landscape
Source: PLoS One. 2024 Feb 8;19(2):e0297942. doi: 10.1371/journal.pone.0297942 (PMC10852327; doi:10.1371/journal.pone.0297942)

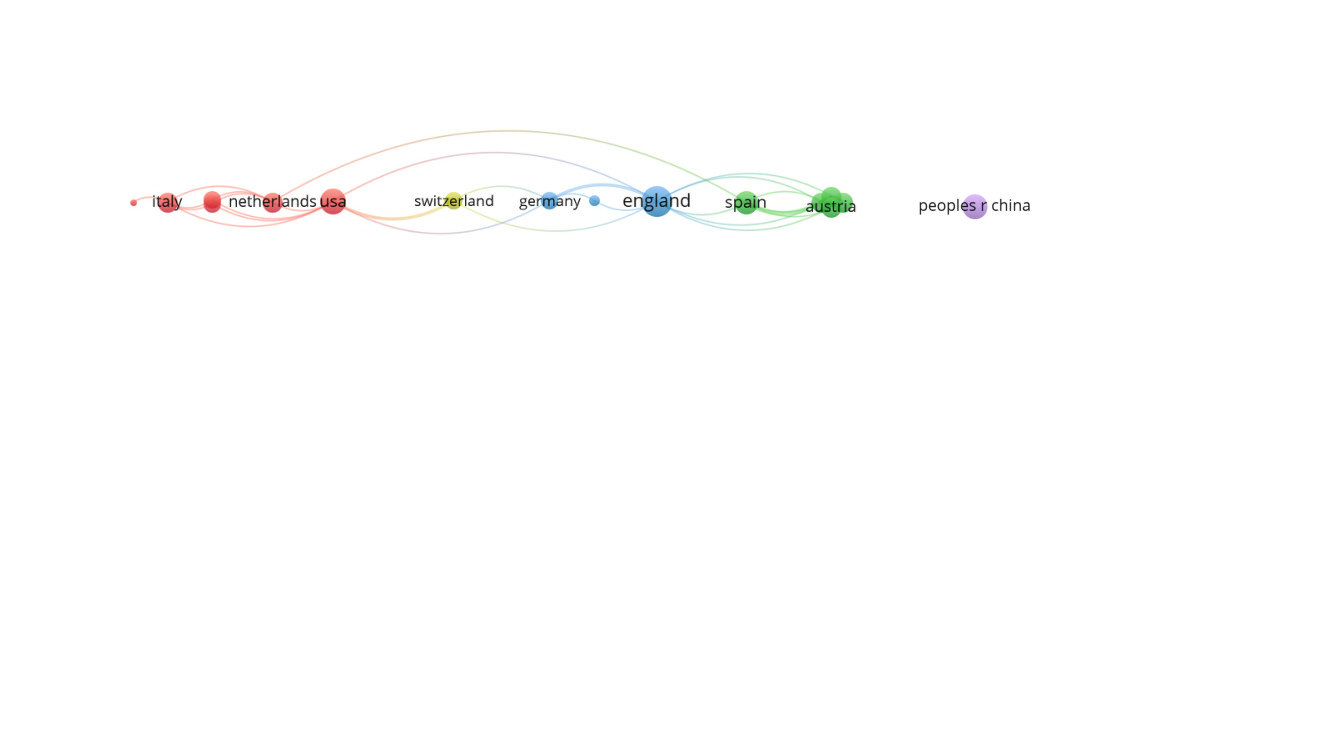

Supplement: S1 Fig — The scale of the cluster represented the articles’ number of corresponding country and the size variation of the line between countries represented the cooperation intensity. Connections of ≥1 were visualized. (TIF) [file pone.0297942.s004.tif]
