# Supplementary material for: Machine perfusion of the liver and in vivo animal models: A systematic review of the preclinical research landscape
Source: PLoS One. 2024 Feb 8;19(2):e0297942. doi: 10.1371/journal.pone.0297942 (PMC10852327; doi:10.1371/journal.pone.0297942)
Supplement: S4 File — (PDF) [file pone.0297942.s008.pdf]

Ex vivo machine perfusion in liver transplantation: a systematic review on characteristics and reporting quality of preclinical animal studies and their adherence to the ARRIVE Guidelines

Citation

Zoltan Czigany, Wenjia Liu, Decan Jiang, Jan Bednarsch, Franziska Alexandra Meister, Anna Mantas, Georg Lurje, Lisa Ernst, Leonie Zieglowski, Dora Tihanyi, Sven Arke Lang, Rene Tolba, Ulf Neumann. Ex vivo machine perfusion in liver transplantation: a systematic review on characteristics and reporting quality of preclinical animal studies and their adherence to the ARRIVE Guidelines. PROSPERO 2021 CRD42021234667 Available from:
https://www.crd.york.ac.uk/prospero/display_record.php?ID=CRD42021234667

Review question [1 change]

1, Which factors influence reporting quality (measured by the ARRIVE score)

in preclinical animal studies on machine perfusion in liver transplantation? AND 2, Which are the most frequently used models, machine perfusion techniques and perfusion conditionings in preclinical machine perfusion studies?

Context and rationale

Since the first introduction in 1963, liver transplantation has evolved as the mainstay of treatment for patients with end stage liver disease. The insufficient availability of donor allografts compared with the growing demand become major issue. An increasing tendency is observed in the numbers of “marginal” liver grafts that have to be accepted for transplantation. Therefore, ex vivo machine perfusion plays an essential role to improve graft preservation and clinical outcomes. Over the past decades dozens of studies have been published to investigate the in vivo effects of machine perfusion in animal models of liver transplantation. In 2010 the ARRIVE (Animal Research: Reporting of In Vivo Experiments) guidelines has been launched to improve transparency and accuracy in bioscience research and studies comparing the reporting standards before and after the ARRIVE guidelines reported controversial results. Our systematic review will provide an overview on the reporting quality, assessing the potential factors influencing scientific and methodological standards in the context of the ARRIVE guidelines in animal studies focusing on liver transplantation combined with ex vivo machine perfusion techniques. This study will also give a comprehensive overview on the used models, techniques and perfusion conditions available in the literature. This will not only provide an accurate and comprehensive snap-shot of the preclinical literature on liver machine perfusion, but also provide an important guidance for the design and execution of subsequent preclinical and clinical trials. Based on these an important and direct link to human liver disease and liver transplantation is provided.

Searches

We will search the following electronic bibliographic databases: PubMed, Web of Science, Embase. The full search strategy is based on the search components: “liver” or "liver transplant" or "hepatic transplant" or “liver transplantation” and "machine perfusion" or “machine preservation”. We will assess publications in English. We will screen reference lists of included studies for additional eligible studies not retrieved by our initial search. We will re-run the searches just before the final analyses to retrieve the most recent studies eligible for inclusion. The search terms will be adapted for use with the corresponding bibliographic databases. Search syntax: (liver transplant OR liver transplantation OR hepatic transplantation) AND (machine perfusion OR machine preservation)

Study designs to be included [1 change]

Inclusion criteria:

The study should include liver transplantation as experimental procedure or model and the machine perfusion as the organ preservation method or study intervention. Since one of the main focus of the review is the evaluation of the reporting quality and the potential factors influencing scientific and methodological standards in context of the ARRIVE guidelines, controlled studies with a separate control group are not necessary.

Exclusion criteria:

We will exclude case studies.

Human disease modelled

liver disease / hepatic disease / end-stage liver disease / acute liver failure

Animals/population [1 change]

Inclusion criteria:

Since the review is in context of the ARRIVE guidelines in animal studies, we will include studies using all kinds of in vivo liver transplantation models (all, species, all strains, all sexes, all ages).

Exclusion criteria:

All studies where the liver is only retrieved and not transplanted into the same (autotransplant) or to a second animal are excluded. Human studies are excluded.

Intervention(s), exposure(s) [1 change]

Inclusion criteria:

Experimental studies which describe liver transplantation combined with ex vivo machine perfusion under anaesthesia (all methods and techniques) are eligible for inclusion. If this is part of a study, the data relevant to the question of this study will be extracted.

Exclusion criteria:

Studies that mention only hepatectomy and machine perfusion without consequential organ transplantation are excluded. Only liver transplant models and cold preservation without machine perfusion technology are likewise excluded.

Comparator(s)/control

Inclusion criteria:

not applicable - In this descriptive systematic review we will focus on the reporting quality of animal studies in the context of the ARRIVE guidelines and the effect or outcome of surgical techniques and experimental setups will not compared with each other, therefore we will include all studies.

Exclusion criteria:

not applicable

Other selection criteria or limitations applied [1 change]

Literature reviews, conference abstracts, systematic reviews, and meta-analysis of experimental studies will be excluded as publication types. Full-text publication in any other language than English will be excluded.

Outcome measure(s) [1 change]

Inclusion criteria:

Graft outcomes, survival, liver injury. Reporting one or more of the outcomes listed here in the trial is not an inclusion criterion for the review. Relevant trials which measured these outcomes but did not report the data at all, or not in a usable format, will be included in the review as part of the narrative.

Exclusion criteria:

We will not exclude studies on the basis of assessed outcome parameters.

Study selection and data extraction [1 change]

Procedure for study selection

Screening will be performed in two phases: initial screening based on title and abstract, followed by full-text screening of the eligible articles for final inclusion. In each phase, two investigators (W.L., D.J.) will independently assess each study. Discrepancies will be resolved through discussion, or by consulting a third investigator (Z.C.) who will also give his judgement.

Prioritise the exclusion criteria

mTitle-Abstract Screening Phase:

1. Human studies

2. Studies where the liver is only retrieved and not transplanted into the same (autotransplant) or to a second animal

3. Only liver transplant models and cold preservation without machine perfusion technology

4. Literature reviews, conference abstracts, systematic reviews, and meta-analysis of experimental studies will be excluded as publication types

5. No full text study

6. Not written English language

Full text-Screening Phase:

As above, with the addition of: full text not published before the final analysis.

Methods for data extraction

Two reviewers will independently extract data from each published study. We first try to extract the required data from text, tables or figures. If these are not reported or unclear, we will try to contact authors by e-Mail (max. 2 attempts).Discrepancies will be resolved through discussion, or by consulting a third investigator (Z.C.) who will also give his judgement.

Data to be extracted: study design

The following date items will be extracted: experimental groups, number of animals per group and survival time after initial organ transplantation. All elements of the ARRIVE guidelines on study design.

Data to be extracted: animal model

The following data items will be extracted: species, strain, sex (male/female), method of liver transplant, details of machine perfusion, anesthetic and analgesic agent used an protocols, follow up, sacrifice

Data to be extracted: intervention of interest

We will describe the following characteristics of the intervention: Hypothermic machine perfusion, Normothermic machine perfusion, Sub-normothermic machine perfusion. Perfusion device and details of perfusion parameters (e.g.

temperature, pressure, time).

Data to be extracted: primary outcome(s)

ARRIVE total Score based on Tihanyi et al. Journal of Surgical Research 2019 (235) 578-590.

Data to be extracted: secondary outcome(s)

Year of publication, Affiliation of the first and last author, Journal, Impact factor in the year of publication, Regions, Institutions, Specialty, Number of funding, Source of funding, Surgical model, Graft type, Experimental animals, categories (genus), strains, Gender of the experimental animals, Applied machine perfusion techniques, Type of organ preservation solution, Cold ischemia time, Warm ischemia time, Most frequently applied follow-up time points, Most frequently applied maximum follow-up, Most frequently applied outcomes, Anesthesia methods.

Data to be extracted: other

none

Risk of bias and/or quality assessment

No risk of bias and/or quality assessment planned.

Strategy for data synthesis

Planned approach

We will provide a synthesis of the findings based on the ARRIVE scores. No metaanalysis will be performed.

Effect measure

not applicable

Effect models

not applicable

Heterogeneity

not applicable

Other

not applicable

Analysis of subgroups or subsets

Subgroup analyses

Subgroup will be analyzed by year of publication, pre- and post-ARRIVE era, affiliation of the first author, journal impact factor, journal endorsement of the ARRIVE guidelines, used animal models, and the presence of funding.

Sensitivity

not applicable

Publication bias

As no meta-analysis is planned, publication bias will not be assessed.

Contact details for further information

Zoltan Czigany

zczigany@ukaachen.de

Organisational affiliation of the review

RWTH Aachen University

https://www.ukaachen.de/kliniken-institute/klinik-fuer-allgemein-viszeral-und-transplantationschirurgie/

Review team members and their organisational affiliations

Dr Zoltan Czigany. RWTH Aachen University

Mr Wenjia Liu. RWTH Aachen University

Mr Decan Jiang. RWTH Aachen University

Dr Jan Bednarsch. RWTH Aachen University

Dr Franziska Alexandra Meister. RWTH Aachen University

Dr Anna Mantas. RWTH Aachen University

Assistant/Associate Professor Georg Lurje. Charité-Universitätsmedizin Berlin

Dr Lisa Ernst. RWTH Aachen University

Dr Leonie Zieglowski. RWTH Aachen University

Dr Dora Tihanyi. RWTH Aachen University

Professor Sven Arke Lang. RWTH Aachen University

Professor Rene Tolba. RWTH Aachen University

Professor Ulf Neumann. RWTH Aachen University

Review type

Animal model review, Pre-clinical animal intervention review

Anticipated or actual start date

01 March 2021

Anticipated completion date

01 July 2021

Funding sources/sponsors

START Program, Faculty of Medicine RWTH Aachen (#23/19)

Grant number(s)

#23/19

Conflicts of interest

Language

English

Country

Germany

Stage of review

Review Ongoing

Subject index terms status

Subject indexing assigned by CRD

Subject index terms

Animals; Animals, Laboratory; Extracorporeal Circulation; Liver Transplantation; Perfusion; Research

Date of registration in PROSPERO

13 March 2021

Date of first submission

07 February 2021

Details of any existing review of the same topic by the same authors

Not applicable

Stage of review at time of this submission

Stage	Started	Completed
Preliminary searches	No	No
Piloting of the study selection process	Yes	No
Formal screening of search results against eligibility criteria	No	No
Data extraction	No	No
Risk of bias (quality) assessment	No	No
Data analysis	No	No

The record owner confirms that the information they have supplied for this submission is accurate and complete and they understand that deliberate provision of inaccurate information or omission of data may be construed as scientific misconduct.

The record owner confirms that they will update the status of the review when it is completed and will add publication details in due course.

Versions

13 March 2021
